# The Influence of the Heat Flux of the Infrared Heater on the Charring Rate of Spruce Wood

**DOI:** 10.3390/polym16182657

**Published:** 2024-09-20

**Authors:** Alena Párničanová, Martin Zachar, Danica Kačíková

**Affiliations:** Department of Fire Protection, Faculty of Wood Sciences and Technology, Technical University in Zvolen, T.G. Masaryka 24, 960 01 Zvolen, Slovakia; zachar@is.tuzvo.sk (M.Z.); kacikova@is.tuzvo.sk (D.K.)

**Keywords:** spruce wood, infrared heater, char layer thickness, charring rate

## Abstract

The study investigates the determination of selected fire properties of spruce wood, specifically the charring rate, using a modified testing method described and registered at the Industrial Property Office of the Slovak Republic PUV 50121-2020, utility model no. 9373. The samples were exposed to a square ceramic infrared heater, FTE-750W, with a power output of 750 W, using which we determined the heat flux as a function of voltage (V). Spruce wood specimens with dimensions of 75 mm × 75 mm × 50 mm (l × w × h) were subjected to thermal exposure under heat fluxes of 10, 15, 20, and 25 kW∙m^−2^. The charring rate was evaluated using two distinct approaches: the first method measured the thickness of the char layer formed after a duration of 1800 s, while the second method was based on reaching a temperature threshold of 300 °C. The findings demonstrated a positive correlation between the thermal load and the charring rate. The charring rates obtained using the first method ranged from 0.2397 to 0.6933 mm∙min^−1^, whereas those derived from the second method varied from 0 to 1.0344 mm∙min^−1^. This suggests that the 300 °C temperature criterion may not be a reliable parameter for calculating the charring rate. The precision of the results was corroborated through numerical simulations.

## 1. Introduction

Spruce wood, whose behavior under thermal load is the subject of this article, is the most widespread natural building material due to its affordability [1]. However, it is a combustible material that contributes to the onset and spread of fires [2]. It can be characterized by fire and technical parameters, which determine the suitability of a specific type of wood for construction purposes [3].

One of the most important fire properties of wood is the charring rate [4]. Several parameters influence it, such as wood density and moisture content [5,6], with moisture slowing down pyrolysis due to its cooling effect—the higher the moisture content, the greater the energy required to evaporate the water and, thus, less energy is available for pyrolysis [7]. The charring rate is also influenced by the type of wood—spruce chars more readily than pine or fir [8]—the direction of burning [9], and the shape of the beam, with a significant difference observed between square profiles and round logs, where the degradation and charring rate of logs is higher [10]. Additionally, external heat flux [4] and the oxygen concentration in the surrounding air are critical factors. Richter et al., 2021 [11], studied the charring and oxidation of particleboard under various conditions, identifying heat flux and ambient oxygen concentration as the main parameters affecting the charring rate and combustion mode between pyrolysis, smoldering, and flaming.

Charring rate values are significant because, according to STN EN 1995-1-2 (Eurocode 5) [12], the charring rate is a crucial factor in calculating the fire resistance of wooden structures, which is of interest to building-safety experts who study the loss of load-bearing capacity of wooden beams and columns in post-flashover conditions [13]. Eurocode 5, Part 1–2 presents several models for calculating the fire resistance of wooden structures. These models are based on the hypothesis that wood chars at temperatures above 300 °C and can no longer withstand any load, as the charred layer has virtually no strength [14]. Besides the charring rate, the char depth is also considered an important parameter for the fire resistance of wooden structures, as it allows the determination of the size of the residual cross-section of the wood, which is used to determine the fire resistance of the wood construction [15].

The charring rate is defined as the rate at which the char depth progresses within the material, or as the time-course of wood degradation due to thermal loading [16]. The charring rate is determined by measuring the depth of charring and the duration of thermal exposure [4]. This parameter is also critical in fire-cause investigations, as highlighted in NFPA 921 (2021) [17], which specifies that the charring rate of wood under laboratory conditions, with heat applied to one side, ranges from 0.17 mm∙min^−1^ to 1.23 mm∙min^−1^. In contrast, EN 1995-1-2 recommends a charring rate of approximately 0.60 mm∙min^−1^ for solid and glued laminated softwood and beech, with the rate decreasing as the wood density increases [18].

It is important to note that the charring rate is not constant during the initial stages of combustion, as it is typically higher at the onset of burning compared to after the char layer has formed. The developing char layer serves as an increasingly effective thermal insulator between the exposed surface and the underlying pyrolyzed wood, leading to a reduction in the charring rate during the early phase of combustion [19].

The aim of this paper was to determine the required voltage at a distance of 30 mm to achieve the selected heat fluxes and to determine the charring rate of spruce wood using two methods: based on the thickness of the charred layer formed over an 1800 s interval and based on the depth at which a temperature of 300 °C was reached, using data obtained from an advanced laboratory testing method. The obtained results were verified by numerical methods.

## 2. Materials and Methods

**Figure 1:**
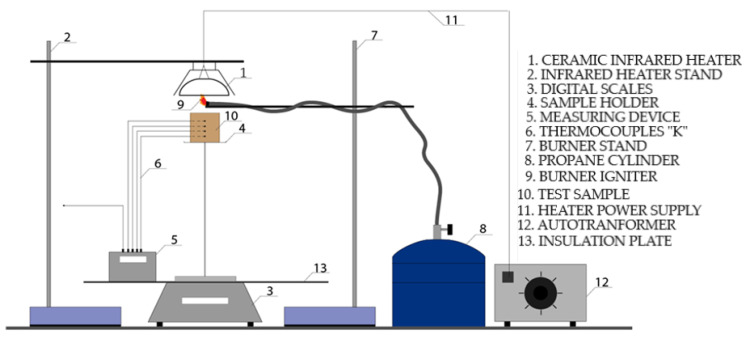
Scheme of laboratory apparatus.

The measurements were conducted using a modified testing method described in Utility Model No. 9373 [20]. The apparatus (Figure 1) consisted of a ceramic infrared heater (type F.T.E.) positioned 30 mm from the sample and a METREL HSN0203 regulatory device (Metrel d.d., Horjul, Slovenia), which was used to regulate and precisely set the voltage at the input terminals of the heater. To achieve the selected heat flux values, it was necessary to determine the heat flux for the ceramic heater. Each heat flux value corresponded to a specific voltage value U.

The heat load values were set to 10, 15, 20, and 25 kW∙m^−2^ to represent different fire intensities and to allow for the study of wood behavior under various thermal stress conditions. The selection of these specific values facilitates a better understanding of the charring process of wood at different levels of thermal load, which is crucial for assessing the fire resistance of wooden structures. Each heat flux value was applied to 10 samples.

The spruce wood samples were prepared from the trunk of Norway spruce (*Picea abies*) harvested from the Ľubická dolina locality, cadastral area Slatinka 595-745 m.a.s.l., stand number 93-00, managed by the University Forestry Enterprise. The type of logging was summer, pre-commercial thinning—intentional, for trees over 50 years old. Air-dried samples were cut tangentially to dimensions of 75 × 75 × 50 mm (l × w × h). Subsequently, selected samples without anatomical defects were sorted into 4 groups, each containing 10 samples (for heat flux loads of 10, 15, 20, and 25 kW∙m^−2^). The thickness of these prepared samples was measured with a Mahr 16ER NO. 4103010 caliper (Mahr GmbH, Göttingen, Germany) at 9 different points to determine the average thickness of the samples. The distance between individual points was 10 mm from the edge of the sample, and the thickness was also measured in the center of the exposed surface (75 × 75 mm). This measurement was used in subsequent steps to determine the thickness of the charred layer. The weight of each sample was then determined.

The absolute moisture content of the samples was determined using Equation (1), which expresses the ratio of the mass of wet wood to the constant weight,

(1) $$w=\frac{m_{v}-m_{0}}{m_{0}}\cdot 100$$

where the following apply:

w—absolute moisture content of the wood (%);
$m_{v}$—weight of wood at a given moisture content (kg);
$m_{0}$—weight of wood at the constant weight (kg).

The density was determined using a computational method, according to Equation (2), which provides the ratio between the mass and the volume of the wood at a specific moisture content,

(2) $$\rho_{w}=\frac{m_{w}}{V_{w}}$$

where the following apply:

$\rho_{w}$—density (kg·m^−3^);
$m_{w}$—weight (kg);
$V_{w}$—volume (m^−3^).

The sample was placed on a stand 30 mm below the infrared heater. At the start of the measurement, a combined thermal load was applied by igniting the sample with a flame and simultaneously exposing it to the heater. The flame of the propane burner was set to a height of 20 mm and positioned 10 mm above the center of the sample. We used a stopwatch to measure the time until ignition and subsequent flaming combustion on the sample’s surface. Once the sample ignited, the gas burner was removed, and the sample was subjected solely to the heater for the remainder of the test. This method allowed us to determine the ignition time of the samples.

The charring rate of the spruce samples was determined based on the temperature profiles occurring in the layers beneath the surface, while the samples were subjected to the ceramic infrared heater. The temperature profile was measured using an ALMEMO 710 (Ahlborn Mess-und Refge-lungstechnik GhbM, Holzkirchen, Germany). A temperature measurement device and four K-type thermocouples, each with a thickness of 0.5 mm, were embedded in the sample at depths of 10 mm (T1), 20 mm (T2), 30 mm (T3), and 40 mm (T4) from the thermally exposed surface. The thickness and subsequent charring rate were then determined based on the time required for each thermocouple (T1, T2, T3, and T4) to reach a temperature of 300 °C, following Equation (3),

(3) $$\beta =\frac{L_{char}}{t}$$

where the following apply:

*β*—charring rate (mm∙min^−1^);
*L_char_*—thickness of the charred layer (mm);
*t*—time of heat loading (min).

After the thermal loading of the samples, the charred layer was manually removed. The samples were then re-measured using a vernier caliper at the same nine locations as before the thermal exposure. The thickness of the charred layer was determined using Equation (4), by calculating the difference between the average thickness of the samples before the experiment and the average thickness after the charred layer was removed,

(4) $$L_{char}={\bar{L}}_{1}-{\bar{L}}_{2}$$

where the following apply:

*L_char_*—thickness of the charred layer (mm);
${\bar{L}}_{1}$—average thickness of the sample before testing (mm);
${\bar{L}}_{2}$—average thickness of the sample after testing (mm).

We verified the accuracy of the results using the following numerical methods:

1. Babrauskas, 2005 [14] calculated the charring rate based on Equation (5):

(5) $$\beta =0.23\cdot q^{0.5}\cdot t^{-0.5}$$

where the following apply:

*β*—charring rate (mm∙min^−1^);
*q*—heat flux (kW∙m^−2^);
*t*—time of heat loading (min).

2. According Lizhong et al., 2008 [21], the charring rate can be calculated based on Equation (6):

(6) $$\beta =\frac{113\cdot q^{0.5}}{\rho \cdot t^{0.3}}$$

where the following apply:

*β*—charring rate (mm∙min^−1^);
*q*—heat flux (kW∙m^−2^);
*t*—time of heat loading (min);
ρ—wood density (kg∙m^−3^).

3. In his study, Butler, 1971 [22] analyzed his own data and some additional results, concluding that the data correspond to Equation (7):

(7) β=0.028·q

where the following apply:

*β*—charring rate (mm∙min^−1^);
*q*—heat flux (kW∙m^−2^).

## 3. Results

### 3.1. Determining the Heat Flux of the Heater Based on Voltage

**Figure 2:**
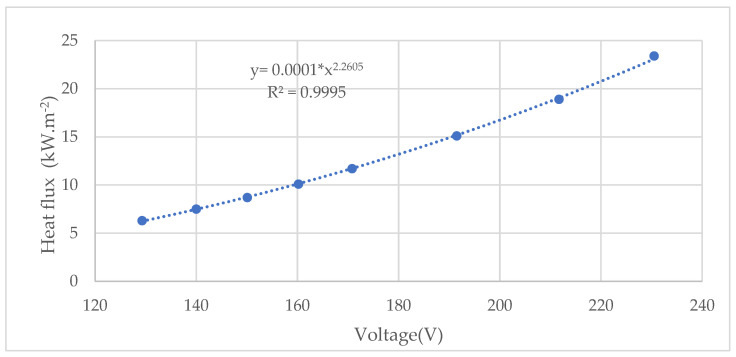
The dependence of heat flux on the output voltage from the autotransformer.

Based on the set voltage values at the output of the autotransformer and the measured voltage data (using a digital multimeter UT71C) and heat flux (using a radiometer), we determined the heat flux of the infrared heater at a distance of 30 mm. A power function graph was constructed based on the measured heat flux values with a reliability equation R^2^ = 0.9995 (Figure 2).

The heat flux of 10 kW∙m^−2^ was calculated at a voltage of 159.21 V, the heat flux of 15 kW∙m^−2^ was determined at a voltage of 190.49 V, the heat flux of 20 kW∙m^−2^ was determined at a voltage of 216.35 V, and the heat flux of 25 kW∙m^−2^ was determined at a voltage of 238.79 V.

### 3.2. Determining the Ignition Time

**Figure 3:**
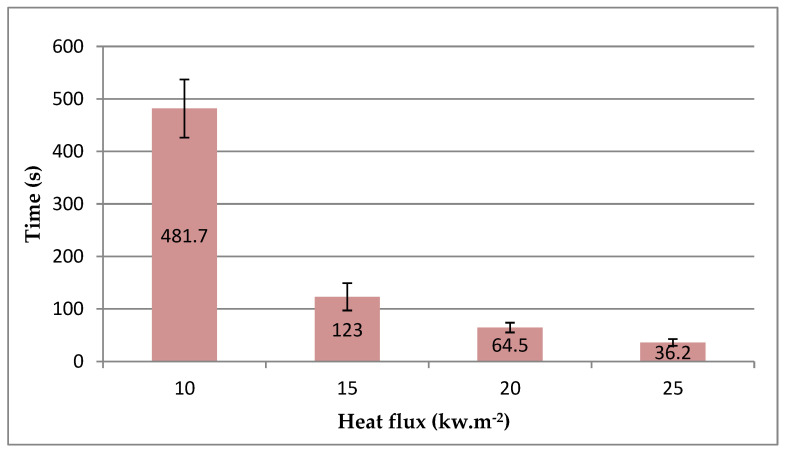
The initiation time as a function of heat flux (error bars represent standard deviation).

The absolute moisture content, calculated based on Equation (1), reached 11.51 ± 0.15%. The sample density calculated according to Equation (2) was 474.3 ± 4.13 kg∙m^−3^. The ignition time, i.e., the time at which the sample ignited, decreased with increasing heat flux (Figure 3).

The samples tested at a heat flux of 10 kW·m^−2^ exhibited the longest average initiation time, reaching 481.7 s. The samples tested at a heat flux of 15 kW·m^−2^ required less than half of that time to initiate, at only 64.5 s. The smallest average measured initiation time was at 25 kW·m^−2^, at 36.2 s, which was approximately half of that at 20 kW·m^−2^, also at 36.2 s.

The initiation time has been the subject of study by many authors, such as Bilbao et al., 2001 [23], who examined the initiation time of Pinus Pinaster samples (110 × 110 × 19 mm). They used the same ignition source as us—a propane flame, which was 10 mm high and placed 10 mm above the sample. Simultaneously, the sample was exposed to selected heat fluxes. The initiation time increased with the increasing heat flux, similar to our experiment. The samples tested at a heat flux of 23.8 kW·m^−2^ exhibited the longest initiation time, at 737 s. The shortest time was recorded at 41.0 kW·m^−2^, at 15 s, approximately half of that recorded at 31.2 kW·m^−2^, at 30 s [23].

Other authors examined the initiation times of beech, oak, and pine samples (100 × 100 × 10 mm) exposed to various heat fluxes in a cone calorimeter. The initiation time for beech at a heat flux of 20 kW·m^−2^ was 865 s, at 30 kW·m^−2^ it decreased to 194 s, and at the highest heat flux, of 50 kW·m^−2^, the initiation time was the shortest, at 46 s. The initiation time for oak at 20 kW·m^−2^ was 621 s, at 30 kW·m^−2^ it decreased to 240 s, and at 50 kW·m^−2^ it was 40 s. The initiation time for pine at 20 kW·m^−2^ was 509 s, at 30 kW·m^−2^ it decreased to 59 s, and at 50 kW·m^−2^ it was 20 s. Spruce, compared to the tested wood species, has a lower initiation time (at 20 kW·m^−2^—36.2 s), igniting sooner [24].

### 3.3. Method 1—The Charring Rate of Spruce Samples Calculated Based on the Value of the Char Layer Thickness

The charring rate of the spruce samples was calculated based on the value of the char layer thickness computed according to Equation (3), which was measured manually. We calculated the average charring rates within the time interval of 0 s to 1800 s according to Equation (3) (Table 1).

The experiment confirmed the hypothesis that the thickness of the charred layer increases with increasing heat flux (Table 1). At heat fluxes of 10, 15, 20, and 25 kW·m^−2^, the thickness of the charred layer reached values of 7.19, 14.34, 17.60, and 20.80 mm, respectively.

**Figure 4:**
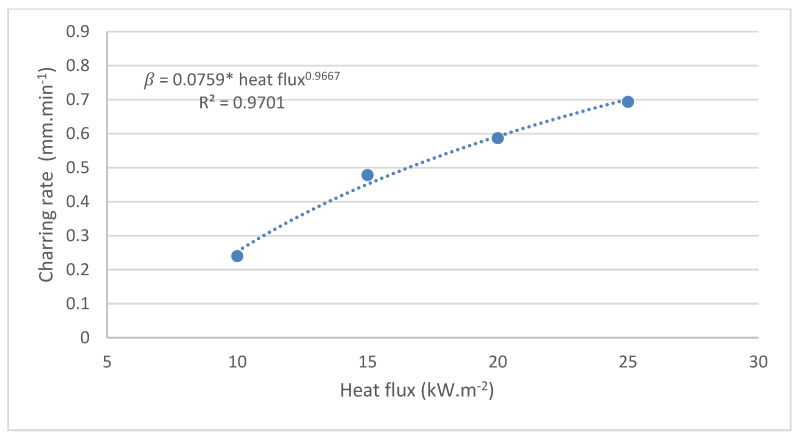
The average charring rate in the time interval of 0 to 1800 s.

The charring rate, with the relationship $\beta =0.0759\cdot q^{0.9667}$, increased with the increasing heat flux (Figure 4). At heat fluxes of 10, 15, 20, and 25 kW∙m^·2^, the charring rate reached values of 0.2397, 0.4780, 0.5867, and 0.6933 mm·min^−1^, respectively.

### 3.4. Method 2—Charring Rate Determined Based on the Charring Thickness Achieved at a Temperature of 300 °C

The charring rate was also determined based on the thickness of the char layer achieved at a temperature of 300 °C, and the time required to reach this char layer thickness. Similarly to the charring rate determined through the calculation, it was confirmed that the charring rate increases with rising heat flux values (Table 2).

During the experiment, a temperature of 300 °C was achieved only at a depth of 10 mm within the samples; no charring occurred at greater depths as the specified temperature was not reached. Similarly, for the heat flux of 10 kW·m^−2^, the charring rate is not provided because the charring temperature of 300 °C was not achieved at a depth of 10 mm within the given time of thermal exposure. At a heat flux of 15 kW·m^−2^, the charring rate was 0.7059 mm·min^−1^. The charring rate reached a higher value of 0.8571 mm·min⁻^1^ at 20 kW·m^−2^. At 25 kW·m^−2^, the charring rate was the highest, measuring 1.0344 mm·min^−1^. These values differ from the charring rates obtained by measuring the char depth with a caliper, suggesting that reaching a temperature of 300 °C might not be a fully relevant metric for calculating the charring rate.

Analyzing the two methods for measuring the charring rate at the selected heat fluxes revealed a statistically significant difference between them (*p* < 0.05), with the second method showing, on average, higher charring rates compared to the first method.

To verify the accuracy of the values, the charring rate was confirmed using numerical methods (Table 3).

By substituting the parameters we determined into Equation (5), we obtained statistically similar results to those achieved by the measurement. The lowest charring rate of 0.2622 mm·min^−1^ was observed at 10 kW·m^−2^, where we measured a similar value of 0.2397 mm·min^−1^. A higher charring rate of 0.3211 mm·min^−1^ was observed at 15 kW·m^−2^, while our measured value was 0.4780 mm·min^−1^. At a heat flux of 20 kW·m^−2^, the charring rate was 0.3708 mm·min^−1^ (our measured charring rate was 0.5867 mm·min^−1^). The highest charring rate, of 0.4145 mm·min^−1^, was achieved at a heat flux of 25 kW·m^−2^ (our measured charring rate was 0.6933 mm·min^−1^). However, some of Babrauskas’s analysis was flawed, since he obtained charring information indirectly from mass loss data and took the residual char density to be zero.

Similarly, statistically similar results were obtained when verifying the charring rates using the calculation method proposed by Lizhong et al., 2008 [21], in which our determined parameters were substituted into the calculation. The lowest charring rate of 0.2716 mm·min^−1^ was observed at 10 kW·m^−2^, where we measured a similar value of 0.2397 mm·min^−1^. A higher charring rate of 0.3326 mm·min^−1^ was recorded at 15 kW·m^−2^, while our measured value was 0.4780 mm·min^−1^; at 20 kW·m^−2^, the charring rate was 0.3841 mm·min^−1^ (our measured charring rate was 0.5867 mm·min^−1^); and the highest charring rate of 0.4294 mm·min^−1^ was achieved at a heat flux of 25 kW·m^−2^ (our measured charring rate was 0.6933 mm·min^−1^). The results of Lizhong’s method demonstrate that the computational model is capable of effectively predicting the charring rate at lower values of time-increasing heat flux. In contrast, the model may be less accurate for materials with extremely low or high densities that were not sufficiently examined within the studied range in the article.

According to Butler, 1971 [22], the results obtained corresponded to Equation (6). By substituting our determined values into the calculation, statistically similar results were obtained to those determined by the measurement. At 10 kW·m^−2^, the charring rate was the lowest, at 0.2800 mm·min^−1^ (our measured value was 0.2397 mm·min^−1^); at 15 kW·m^−2^, the charring rate increased to 0.4200 mm·min^−1^ (our measured value was 0.4780 mm·min^−1^); at 20 kW·m^−2^, the charring rate was 0.5600 mm·min^−1^ (our measured value was 0.5867 mm·min^−1^); and the highest charring rate, of 0.7000 mm·min^−1^, was achieved at a heat flux of 25 kW·m^−2^ (our measured value was 0.6933 mm·min^−1^). This method provides simple initial estimates of the charring rate based on the heat flux; however, the model does not account for variations in material density or composition, which can significantly influence the charring rate. Materials with very low or very high density may exhibit different charring characteristics that are not captured by the model.

The charring rates obtained by the first measurement method were similar to existing standards cited in the literature, whereas the charring rates calculated based on reaching the charring temperature at a certain depth did not statistically agree with the values obtained by measuring the thickness of the charred layer using a sliding caliper, similarly to our measured values, when compared with the values calculated by numerical methods. The assertion that reaching a temperature of 300 °C may not be a relevant parameter in calculating charring rates was confirmed by comparing the results of the numerical methods.

**Figure 5:**
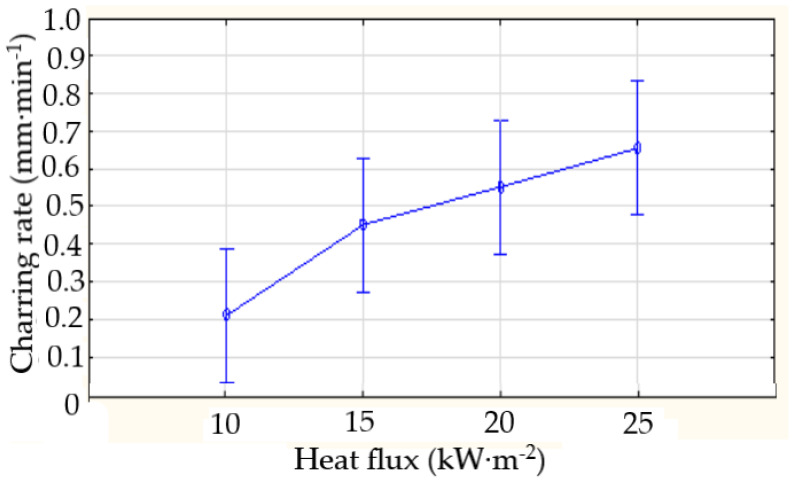
Standard deviation of the charring rate values from Table 3.

Based on the analysis using STATISTICA 12.0.1133.23 64 software (Figure 5), we found that the F value reached 5.1022, and the *p*-value was 0.01147. This result indicates statistical significance because the differences between the groups were statistically significant at a significance level of *p* < 0.05. Similarly, since the F value exceeded the critical value, we can also acknowledge the existence of a statistical difference between the observed groups. These results confirm our previous claims and provide evidence of a statistically significant relationship between the variables in our analysis.

## 4. Discussion

Other authors have also investigated the rate of charring. Kamenická et al., 2018 [25], analyzed the charring rates of wooden elements with various cracks, using samples with zero moisture content and dimensions of 60 × 100 × 100 mm subjected to a heat flux of 25 kW·m^−2^ in a cone calorimeter for 40 min. They found that cracks can influence wood charring at the crack site and, in the case of multiple cracks in the cross-section, affect the entire cross-section’s charring. The charring rate of samples without cracks was approximately 0.7000 mm·min^−1^, which is comparable to the value obtained in our measurements, specifically, 0.6933 mm·min^−1^. The samples with 6 × 24 mm cracks achieved a charring rate of approximately 0.9000 mm·min^−1^, while those with larger 3 × 4 × 32 mm cracks reached a charring rate of approximately 1.1000 mm·min^−1^. The authors concluded that cracks can affect wood charring at the crack site and, in the case of multiple cracks in the cross-section, the entire cross-section.

In thermally loaded samples of Japanese larch (*Larix kaempferi*), a charring rate of mm·min^−1^ was determined, which is comparable to the charring rate specified in the standard EN 1995-1-2 2004 (0.80 mm·min^−1^) [26], which is consistent with the values we measured at 25 kW·m^−2^ (0.6933 mm·min^−1^).

Hakkarainen, 2002 [27], measured the charring rate of laminated wooden elements in a large room fire. After 40 min, a char depth of 30 mm formed on the ceiling, with the average charring rate ranging from 0.6000 to 0.7500 mm·min^−1^, which is similar to the values we measured (0.6933 mm·min^−1^).

Lawson et al. (1952) [28] tested Douglas fir beams according to ISO 834 [29] for 29 min and found an average charring rate of 0.6350 mm·min^−1^, which is comparable to the values we measured (0.6933 mm·min^−1^) at a heat flux of 25 kW·m^−2^.

Based on experiments, Babrauskas, 2005 [14], found that in extensive room fires, heavy wooden or similar elements without gaps or joints char at similar rates as in furnace tests, at approximately 0.5000–0.8000 mm·min^−1^, which are similar to the values we obtained at 25 kW·m^−2^ (0.6933 mm·min^−1^). The author suggests that the charring rate in real fires should not exceed these test values.

## 5. Conclusions

We determined the charring rate of spruce wood based on the thickness of the char layer formed over a time interval of 1800 s using a modified testing method. Samples were thermally loaded using a ceramic infrared heater, FTE-750W (Ceramix Ltd., Ballydehob, Ireland), for which we determined the heat flux depending on the voltage (V). At a heat flux of 10 kW·m^−2^, the voltage reached a value of 159.21 V, a slightly higher value was reached at 15 kW·m^−2^ with 190.49 V, at 20 kW·m^−2^, the voltage was 216.35 V, and at 25 kW·m^−2^, it corresponded to 238.79 V. These voltage values exhibit a linear increase in heat flux.

The experiment demonstrated that the ignition time decreases as the heat flux increases, while the charring rate exhibits a positive correlation with rising heat flux. The charring rate was assessed using two distinct methods: one involved measuring the thickness of the char layer with a vernier caliper, and the other determined the thickness corresponding to a temperature of 300 °C.

To verify the results, we used the numerical method according to Babrauskas, 2005, Lizhong et al., 2008, and Butler, 1971, through which we obtained similar results to those obtained with the measurement of the char layer thickness using a vernier caliper. However, the measurements based on reaching a temperature of 300 °C showed different charring rate values, indicating that achieving a temperature of 300 °C may not be considered a relevant parameter in charring rate calculation. The charring rate values could also have differed because the measured temperature around the thermocouples may have reached higher values, possibly due to the faster heating of the metal from which the thermocouples were made compared to wood. This could also have resulted in higher charring rate values. Similarly, our measured charring rate values may be higher than those in the Eurocode, as the Eurocode values may be overdesigned to provide greater fire safety. It is assumed that a higher charring rate leads to a faster reduction in effective cross-section. We hypothesize that lower charring rate values provide a more reliable and safer construction that withstands fires while offering better stability in case of fire hazards.

## Figures and Tables

**Table 1:** The charring rate within the time interval of 0 to 1800 s.

Heat Flux (kW·m^−2^)	Thickness of the Sample before Testing ($\bar{L}$_1_) (mm)	Thickness of the Sample after Testing ($\bar{L}$_2_) (mm)	Thickness of the Charred Layer (*L_char_*) (mm)	Charring Rate (mm·min^−1^)
10	50.87 ± 0.13	43.68 ± 0.38	7.19 ± 0.21	0.2397
15	51.28 ± 0.20	36.94 ± 0.18	14.34 ± 0.51	0.4780
20	51.01 ± 0.97	33.41 ± 0.29	17.60 ± 0.59	0.5867
25	51.17 ± 0.13	30.37 ± 0.71	20.80 ± 0.27	0.6933

**Table 2:** The charring rates of the samples in time intervals at a depth of 10 mm.

Heat Flux (kW·m^−2^)	The Temperature within the Sample at a Depth of 10 mm (°C)	Time of Heat Loading (s)	The Charring Rate in the Time Interval of 0 to 850 s (mm·min^−1^)
10	-	-	-
15	300	850	0.7059
20	300	700	0.8571
25	300	580	1.0344

**Table 3:** Comparison of charring rates calculated using numerical methods.

Heat Flux (kW·m^−2^)	*β* Based on Reaching 300 °C	*β* Measured Using Caliper	Calculation of *β* According to Equation (5)	Calculation of *β* According to Equation (6)	Calculation of *β* According to Equation (7)
10	-	0.2397	0.2622	0.2716	0.2800
15	0.7059	0.4780	0.3211	0.3326	0.4200
20	0.8571	0.5867	0.3708	0.3841	0.5600
25	1.0344	0.6933	0.4145	0.4294	0.7000

## Data Availability

The original contributions presented in the study are included in the article, further inquiries can be directed to the corresponding author.
